# Transcriptional, chromatin, and metabolic landscapes of LDHA inhibitor–resistant pancreatic ductal adenocarcinoma

**DOI:** 10.3389/fonc.2022.926437

**Published:** 2022-08-02

**Authors:** Parmanand Malvi, Vipin Rawat, Romi Gupta, Narendra Wajapeyee

**Affiliations:** ^1^ Department of Biochemistry and Molecular Genetics, University of Alabama at Birmingham, Birmingham, AL, United States; ^2^ O’Neal Comprehensive Cancer Center, University of Alabama at Birmingham, Birmingham, AL, United States

**Keywords:** cancer metabolism, LDHA, metabolomics, transcriptomics, chromatin accessibility

## Abstract

Metabolic reprogramming, due in part to the overexpression of metabolic enzymes, is a key hallmark of cancer cells. Lactate dehydrogenase (LDHA), a metabolic enzyme that catalyzes the interconversion of lactate and pyruvate, is overexpressed in a wide variety of cancer types, including pancreatic ductal adenocarcinoma (PDAC). Furthermore, the genetic or pharmacological inhibition of LDHA suppresses cancer growth, demonstrating a cancer-promoting role for this enzyme. Therefore, several pharmacological LDHA inhibitors are being developed and tested as potential anti-cancer therapeutic agents. Because cancer cells are known to rapidly adapt and become resistant to anti-cancer therapies, in this study, we modeled the adaptation of cancer cells to LDHA inhibition. Using PDAC as a model system, we studied the molecular aspects of cells resistant to the competitive LDHA inhibitor sodium oxamate. We performed unbiased RNA-sequencing (RNA-seq), assay for transposase-accessible chromatin with sequencing (ATAC-seq), and metabolomics analyses of parental and oxamate-resistant PDAC cells treated with and without oxamate to identify the transcriptional, chromatin, and metabolic landscapes of these cells. We found that oxamate-resistant PDAC cells were significantly different from parental cells at the levels of mRNA expression, chromatin accessibility, and metabolites. Additionally, an integrative analysis combining the RNA-seq and ATAC-seq datasets identified a subset of differentially expressed mRNAs that directly correlated with changes in chromatin accessibility. Finally, functional analysis of differentially expressed metabolic genes in parental and oxamate-resistant PDAC cells treated with and without oxamate, together with an integrative analysis of RNA-seq and metabolomics data, revealed changes in metabolic enzymes that might explain the changes in metabolite levels observed in these cells. Collectively, these studies identify the transcriptional, chromatin, and metabolic landscapes of LDHA inhibitor resistance in PDAC cells. Future functional studies related to these changes remain necessary to reveal the direct roles played by these changes in the development of LDHA inhibitor resistance and uncover approaches for more effective use of LDHA inhibitors in cancer therapy.

## Introduction

Metabolic reprogramming is a key hallmark of cancer cells and represents a therapeutic vulnerability in various cancer types (1–3). Notably, a number of enzymes and transporters that promote glycolysis and glucose metabolism are overexpressed in cancer cells relative to normal cells (4, 5). One such enzyme, lactic acid dehydrogenase A (LDHA), is known to be overexpressed in several cancer types and associated with poor prognosis (6–10).

LDHA encodes the A subunit of lactate dehydrogenase, a metabolic enzyme that catalyzes the interconversion of lactate and pyruvate (11, 12). The LDHA dependency of cancer cells has been demonstrated in several xenograft and genetically engineered mouse models, in which the genetic ablation of LDHA significantly reduces tumor growth (13–15). These studies prompted the development and testing of LDHA inhibitors (LDHAi) for cancer treatment (16–19). One such inhibitor, sodium oxamate (hereafter referred to as oxamate), is a pyruvate analog and thus a competitive LDHAi that halts lactate production through LDHA inhibition (19). Therefore, similar to the genetic inhibition of LDHA, oxamate treatment also results in tumor suppression due to the loss of LDHA activity (19, 20).

Cancer cells have been shown to depend upon various nutrients as either energy source or building blocks and these are shown to be necessary for their survival (2). In particular, depending upon whether cancer cells utilize primarily lipid or glucose they are categorized as lipogenic or glycolytic (21–23). A previous study used metabolic profiling to stratify pancreatic ductal adenocarcinoma (PDAC) into subtypes with distinct sensitivities to metabolic inhibitors (23). This prior study identified two major PDAC classes: those that are sensitive to lipogenesis inhibitors, such as stearoyl CoA desaturase (SCD) inhibitors, and those that are sensitive to glycolytic inhibitors, such as oxamate (23). LDHA is overexpressed in PDAC and predicts poor prognosis (24, 25), and LDHA inhibition has been shown to suppress PDAC growth and progression (26, 27).

In this study, we modeled the mechanism of resistance to the LDHAi oxamate, aiming to understand the mechanisms underlying cancer cell adaptation to glycolysis and LDHA inhibition, which may lead to improved glycolysis targeting and LDHAi–based cancer therapies. We used a glycolytic PDAC cell line MIAPaCa2 as a model system and employed several integrated and unbiased approaches, including RNA-sequencing (RNA-seq)-based mRNA profiling, assay for transposase-accessible chromatin with sequencing (ATAC-seq)-based chromatin accessibility profiling, and large-scale untargeted global metabolomics analysis to identify the transcriptional, chromatin, and metabolic features of oxamate-sensitive and oxamate-resistant PDAC cells and characterize their responses to oxamate treatment.

## Results

### Transcriptome-wide RNA-sequencing analysis identifies distinct mRNA expression profiles between oxamate response and oxamate resistance in PDAC cells

**Figure 1:**
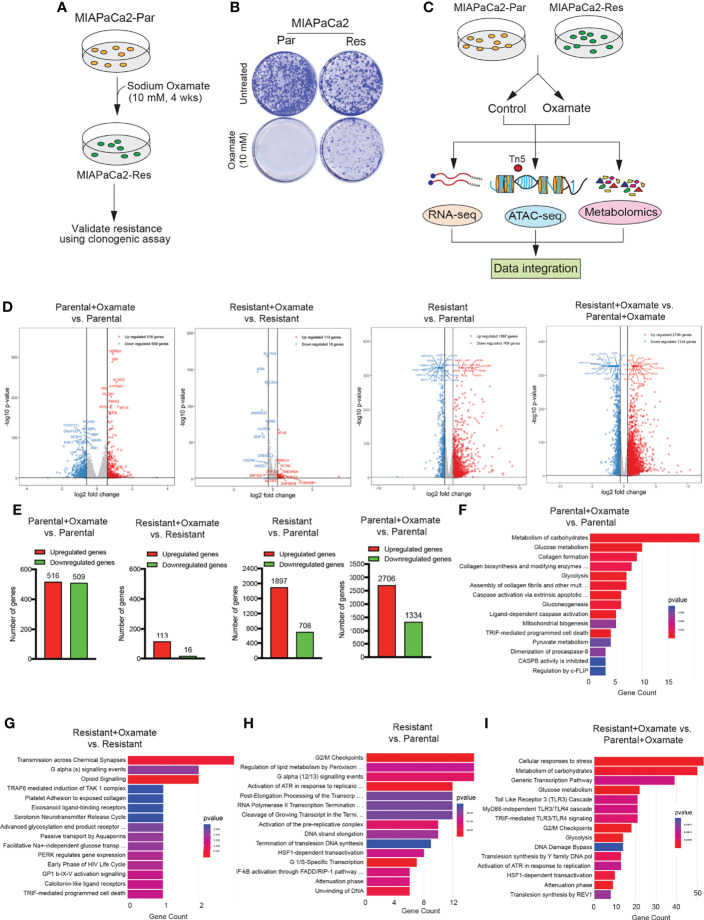
Generation of sodium oxamate–resistant cell lines and RNA-sequencing to identify differentially expressed mRNAs. **(A)** Schematic showing the steps used to generate and validate sodium oxamate–resistant MIAPaCa2 cells. **(B)** parental (Par) or sodium oxamate–resistant (Res) MIAPaCa2 cells were treated without or with 10 mM sodium oxamate (oxamate) for 2 weeks, followed by clonogenic assay. Representative colonies showing oxamate resistance are shown. **(C)** Schematic showing the pipeline for multiple unbiased omics approaches to determine the transcriptional, chromatin, and metabolic profiles of parental and oxamate-resistant MIAPaCa2 cells. **(D)** Volcano plot showing differentially expressed genes (up- or downregulated) in the indicated comparisons. The top 10 up- and downregulated genes based on p-values are also labeled. **(E)** Bar diagram showing the number of up- or downregulated mRNAs (genes) in the indicated comparisons. **(F–I)** Reactome Pathway Analysis showing key biological pathways associated with the differentially expressed mRNAs in the indicated comparisons.

To identify alterations that occur in pancreatic cancer cells able to survive LDHA inhibition, we treated the oxamate-sensitive glycolytic PDAC cell line MIAPaCa2 (parental cells) with 10 mM oxamate for 4 weeks to generate an oxamate-resistant PDAC cell line (**Figure 1A**). MIAPaCa2 is a glycolytic cell line as observed by a previous study used metabolic profiling to stratify pancreatic ductal adenocarcinoma (PDAC) into subtypes with distinct sensitivities to metabolic inhibitors (23). Oxamate resistance in MIAPCa2 cells that survived oxamate treatment was confirmed using a clonogenic assay (**Figure 1B**). We then performed a series of large-scale analyses, including RNA-seq, ATAC-seq, and untargeted global metabolomics, to uncover the differences between parental and oxamate-resistant MIAPaca2 cells and their responses to oxamate treatment (**Figure 1C**).

We first performed RNA-seq to monitor changes in mRNA expression in parental and oxamate-resistant MIAPaCa2 cells treated with either vehicle or oxamate. RNA-seq analysis showed that the treatment of parental MIAPaCa2 cells with oxamate led to significant changes in mRNA expression, evidenced by the identification of 1025 differentially expressed mRNAs following oxamate treatment compared with vehicle treatment (516 upregulated and 509 downregulated mRNAs; **Figures 1D**, **E** and **Table S1**). However, oxamate-resistant MIAPaCa2 cells were largely transcriptionally non-responsive to oxamate treatment, with only 129 genes identified as differentially expressed following oxamate treatment compared with vehicle treatment (113 upregulated and 16 downregulated mRNAs; **Figures 1D**, **E** and **Table S2**). Consistent with the finding that parental and oxamate-resistant MIAPaCa2 cells display significantly different mRNA expression profiles, we observed 2605 differentially expressed mRNAs (1897 upregulated and 708 downregulated mRNAs) in oxamate-resistant MIAPaCa2 cells compared with parental MIAPaCa2 cells (**Figures 1D**, **E** and **Table S3**). The differences were even more pronounced when both cell lines were treated with oxamate, resulting in 4040 differentially expressed mRNAs (2706 upregulated and 1334 downregulated mRNAs) in oxamate-resistant MIAPaCa2 cells compared with parental MIAPaCa2 cells (**Figures 1D**, **E** and **Table S4**). Reactome Pathway Analysis of differentially regulated mRNAs across various comparisons revealed the enrichment of many distinctly regulated biological pathways (**Figures 1F–** **I**). Collectively, these results demonstrate that parental and oxamate-resistant PDAC cells show significantly different transcriptional profiles and significantly different responses to oxamate, resulting in the enrichment of distinct biological pathways.

### ATAC-seq reveals significant differences in chromatin accessibility patterns between oxamate response and resistance in PDAC cells

**Figure 2:**
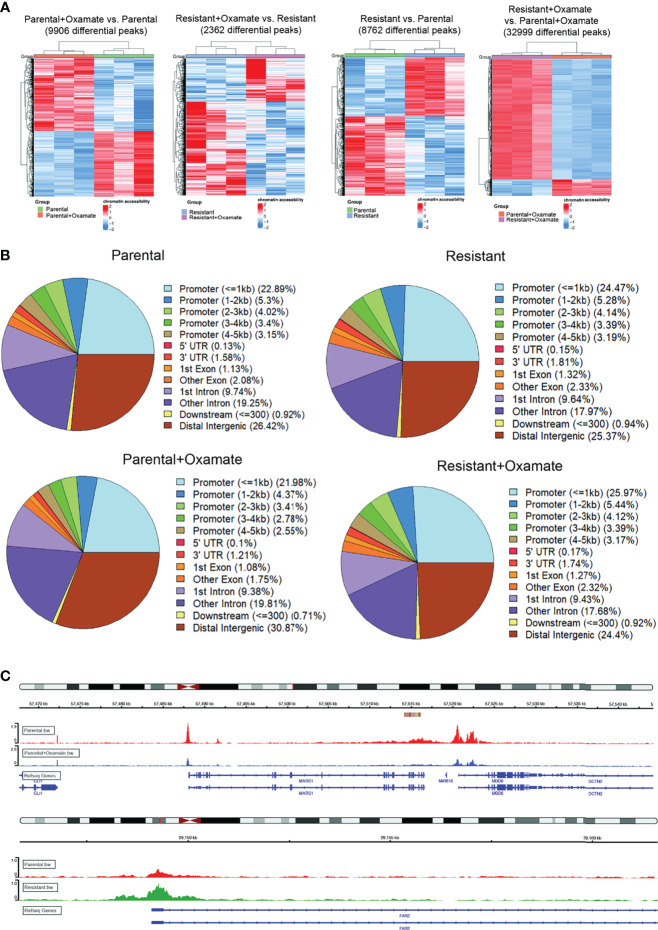
ATAC-seq analysis of parental and oxamate-resistant MIAPaCa2 cells. **(A)** Heatmaps showing differential genomics regions with increased or decreased chromatin accessibility based on ATAC-seq identified in the indicated comparisons. **(B)** Pie-chart for the indicated samples mapping the locations of annotated peaks identified by ATAC-seq. **(C)** IGV tracks for the indicated genomic regions for the indicated conditions.

The upregulation or downregulation of mRNA expression can correlate with increased or reduced chromatin accessibility, respectively (28). Therefore, we measured changes in chromatin accessibility in parental and oxamate-resistant MIAPaCa2 cells treated with and without oxamate using ATAC-seq. The ATAC-seq results showed that parental MIAPaCa2 cells treated with oxamate underwent a 4-fold increase in chromatin accessibility changes (9906 differentially expressed peaks) compared with oxamate-resistant MIAPaCa2 cells treated with oxamate (2362 differentially expressed peaks) (**Figures 2A**, **B**). These results were consistent with the larger number of differentially expressed mRNAs observed in oxamate-treated parental MIAPaCa2 cells than in oxamate-treated oxamate-resistant MIAPaCa2 cells. Similarly, parental MIAPaCa2 cells treated with oxamate were significantly different from oxamate-resistant MIAPaCa2 cells treated with oxamate and showed 32,999 changes in chromatin accessibility. A greater than 2-fold increase in mRNAs were differentially expressed in the comparison between parental MIAPaCa2 cells and oxamate-resistant MIAPaCa2 cells than in the comparison between vehicle-treated and oxamate-treated parental cells. However, a similar number of changes in chromatin accessibility was observed between vehicle- (8762 differential peaks) and oxamate-treated parental MIAPaCa2 cells (9906 differential peaks) (**Figure 2A**). As expected, the Integrative Genomics Viewer (IGV) tracks were consistent with the expected changes in chromatin accessibility; two such examples, for the genomic regions encoding MARS1 and FAR2, under the indicated sample conditions are shown in **Figure 2C**. Under all conditions, a large majority of chromatin accessibility changes were associated with promoter regions, including approximately 35% in oxamate-treated parental MIAPaCa2 cells, approximately 38% in vehicle-treated parental MIAPaCa2 cells, approximately 40% in vehicle-treated oxamate-resistant MIAPaCa2 cells, and approximately 42% in oxamate-treated oxamate-resistant MIAPaCa2 cells (**Figure 2B**). Collectively, these results demonstrate that similar to the differences in mRNA expression patterns, parental and oxamate-resistant MIAPaCa2 cells demonstrate distinct chromatin accessibility profiles.

### Integration of RNA-seq and ATAC-seq data reveals direct correlations between differential mRNA expression and changes in chromatin accessibility

**Figure 3:**
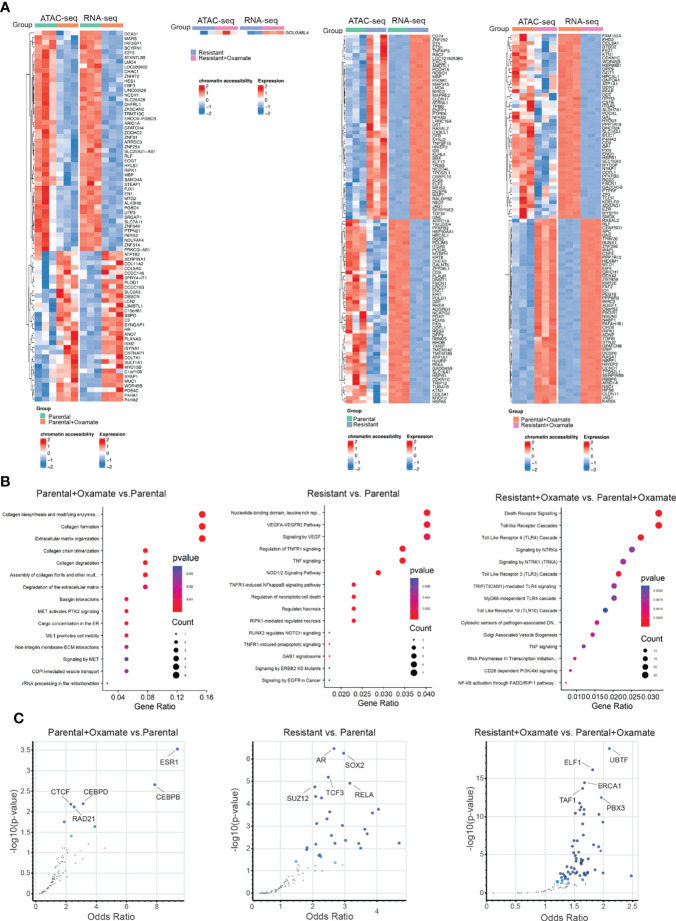
RNA-seq and ATAC-seq integrative analysis reveals genes that display coherence between changes in chromatin accessibility and mRNA expression levels. **(A)** Heatmap for the top 100 (50 upregulated or with increased chromatin accessibility and 50 downregulated or with reduced chromatin accessibility) genes showing similar patterns in both the ATAC-seq and RNA-seq analyses. **(B)** Reactome Pathway Analysis showing enriched biological pathways for genes that display coherence between changes in chromatin accessibility and mRNA expression levels. **(C)** Enrichr analysis using ENCODE and ChEA for identifying potential regulatory transcription factors for differentially regulated metabolic genes identified in the indicated comparisons.

To identify differentially expressed mRNAs associated with changes in chromatin accessibility, such as upregulated mRNAs in areas of increased chromatin accessibility and vice versa, we integrated the RNA-seq and ATAC-seq data across various comparisons. Of 1025 differentially expressed mRNAs identified in the comparison between vehicle- and oxamate-treated parental MIAPaCa2 cells, only 79 mRNAs (approximately 8%) were associated with similar directional changes for both mRNA expression and chromatin accessibility (**Figure 3A**). Only 1 of 129 (less than 1%) differentially expressed mRNAs in the comparison between vehicle- and oxamate-treated oxamate-resistant cells displayed similar directional changes for both mRNA expression and chromatin accessibility (**Figure 3A**). Furthermore, 310 of 2605 differentially expressed mRNAs in the comparison between parental and oxamate-resistant cells (approximately 12%) showed similar directional changes for both mRNA expression and chromatin accessibility (**Figure 3A**). Of 4040 differentially expressed mRNAs identified in the comparison between oxamate-treated parental and oxamate-resistant MIAPaCa2 cells, 193 (approximately 5%) showed similar directional changes for both mRNA expression and chromatin accessibility. Collectively, the integrated analysis revealed a subset of mRNAs with expression changes that occurred concurrently and in the same direction as changes in chromatin accessibility.

Next, we performed Reactome Pathway Analysis for only those differentially expressed mRNAs associated with similar directional changes in chromatin accessibility. The integrated analysis of changes in mRNA expression and chromatin accessibility revealed no specific biological pathways enriched in oxamate-treated oxamate-resistant MIAPaCa2 cells compared with vehicle-treated oxamate-resistant MIAPaCa2 cells, indicating that these cells were largely non-responsive to oxamate treatment. However, various distinct biological pathways were enriched in the other comparisons, as shown in **Figure 3B**.

We then aimed to identify transcription factors potentially involved in the regulation of mRNA expression of genes associated with similar directional changes in chromatin accessibility. To this end, we used the Enrichr platform together with the Encyclopedia of DNA elements (ENCODE) and chromatin immunoprecipitation (ChIP) Enrichment Analysis (ChEA) to identify consensus transcription factors using the ChIP-X option (29). This particular setting of Enrichr allows for the identification of consensus target genes regulated by transcription factors found in the ENCODE and ChEA databases. For transcription factors with data from multiple experiments, set intersection was applied to obtain consensus. For each comparison, multiple transcription factors were identified as potentially direct transcriptional regulators for differentially expressed mRNAs (**Figure 3C** and **Tables S5**–**S7**). In particular, in the comparison between parental and oxamate-resistant MIAPaCa2 cells, androgen receptor (AR), SRY-box transcription factor 2 (SOX2), transcription factor 3 (TCF3), SUZ12, and RELA were identified as the top-most significantly enriched transcription factors. In the comparison between vehicle- and oxamate-treated parental MIAPaCa2 cells, estrogen receptor 1 (ESR1), CCAAT enhancer–binding protein beta and delta (CEBPB and CEBPD, respectively), CCCTC-binding factor (CTCF), and RAD21 were identified as the top-most significantly enriched transcription factors. In the comparison between oxamate-treated parental and oxamate-resistant MIAPaCa2 cells, upstream binding transcription factor (UBTF), E74-like ETS transcription factor 1 (ELF1), breast cancer 1 (BRCA1), TATA-box–binding protein–associated factor 1 (TAF1), and PBX homeobox 3 (PBX3) were identified as the top-most enriched transcription factors.

### Untargeted global metabolomics analysis identifies metabolic patterns in parental and oxamate-resistant PDAC cells

**Figure 4:**
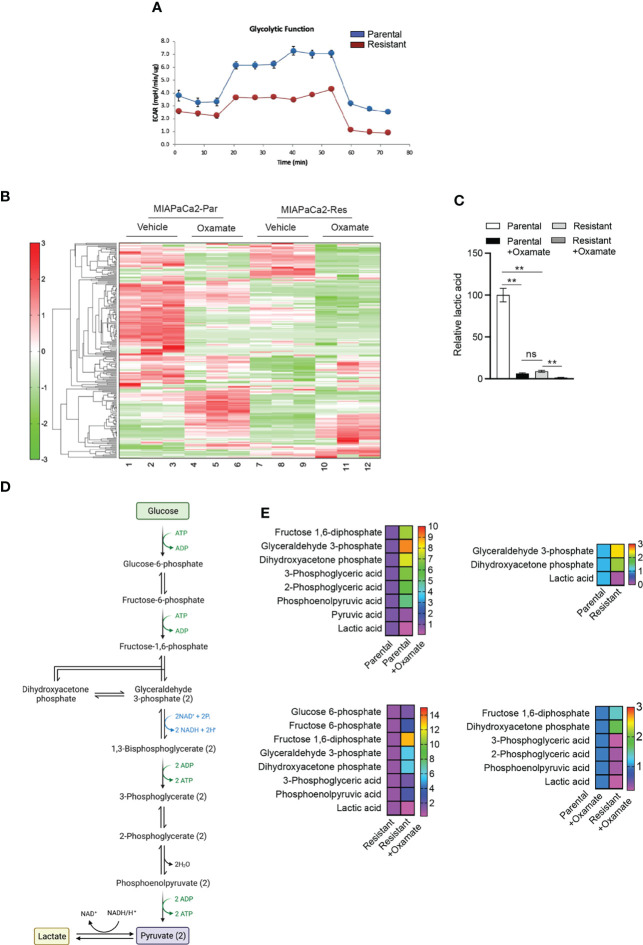
Metabolic profiling of parental and oxamate-resistant cells treated with or without sodium oxamate. **(A)** Seahorse analysis of the glycolysis stress test measuring the glycolytic functions of parental and oxamate-resistant cells. Extracellular acidification rates for parental and oxamate-resistant MIAPaCa2 cells are shown. **(B)** Heatmap showing differences in metabolite levels among parental (Par) and oxamate-resistant (Res) MIAPaCa2 cells treated with vehicle or oxamate. **(C)** Relative lactic acid levels in parental and oxamate-resistant cells treated with and without oxamate are shown. **(D)** Schematic showing glycolysis and the step for pyruvate to lactate conversion. **(E)** Changes in the indicated metabolites involved in glycolytic pathways under the indicated conditions.

We next decided to examine metabolic changes in parental and oxamate-resistant MIAPaCa2 cells treated with and without oxamate. We first performed a Seahorse analysis to measure glycolytic functions in parental and oxamate-resistant MIAPaCa2 cells. We found that oxamate treatment resulted in reduced extracellular acidification in oxamate-resistant MIAPaCa2 cells compared with parental MIAPaCa2 cells, suggesting that oxamate-resistant cells showed inhibition of glycolysis (**Figure 4A**). This finding is consistent with the RNA-seq and ATAC-seq data results (**Figure 1D** and **Figure 3A**), which showed that oxamate-resistant cells were largely non-responsive to oxamate treatment at the mRNA expression and chromatin accessibility levels, respectively.

**Figure 5:**
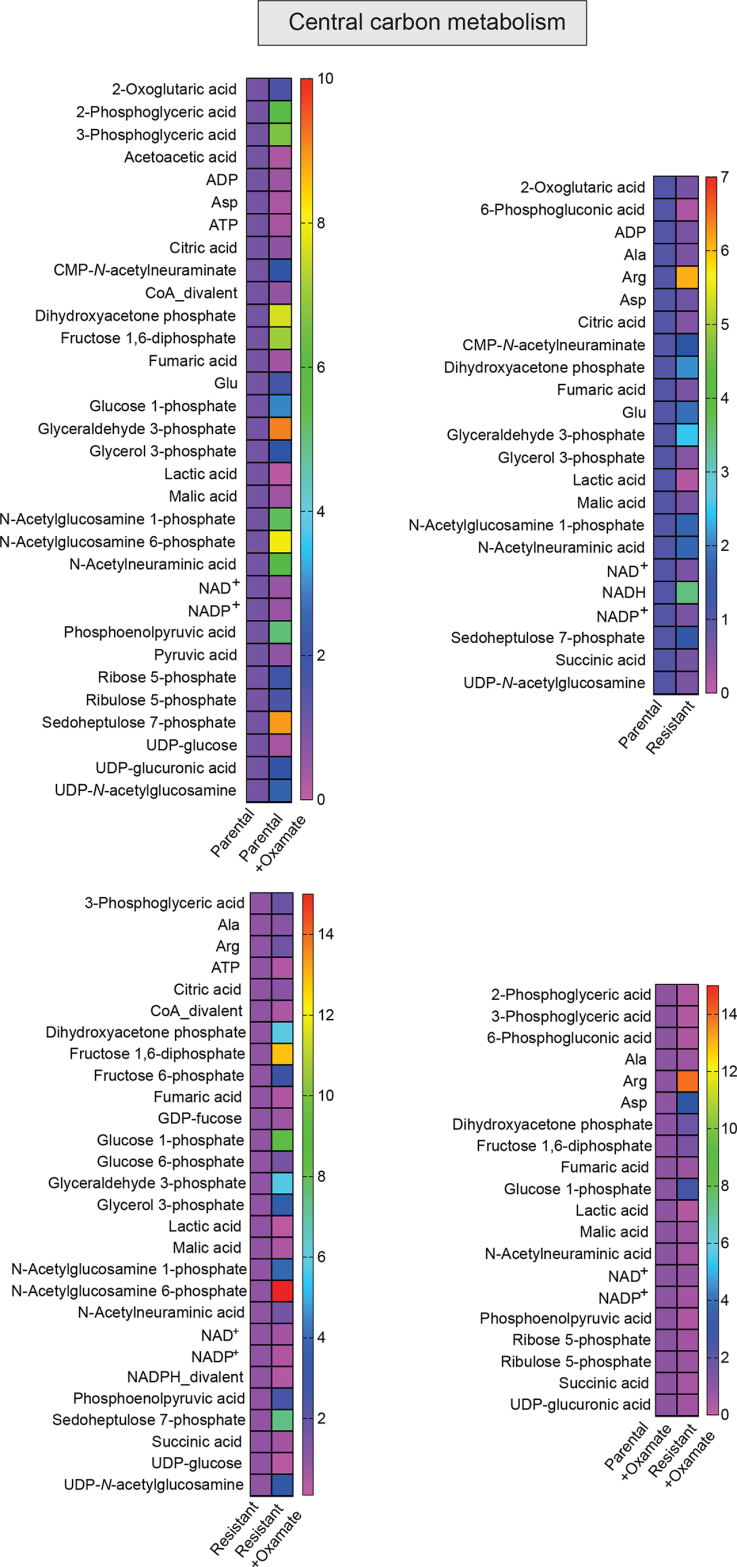
Metabolites generated during central carbon metabolism in parental and oxamate-resistant cells treated with or without sodium oxamate. Changes in the indicated metabolites generated during central carbon metabolism under the indicated conditions.

**Figure 6:**
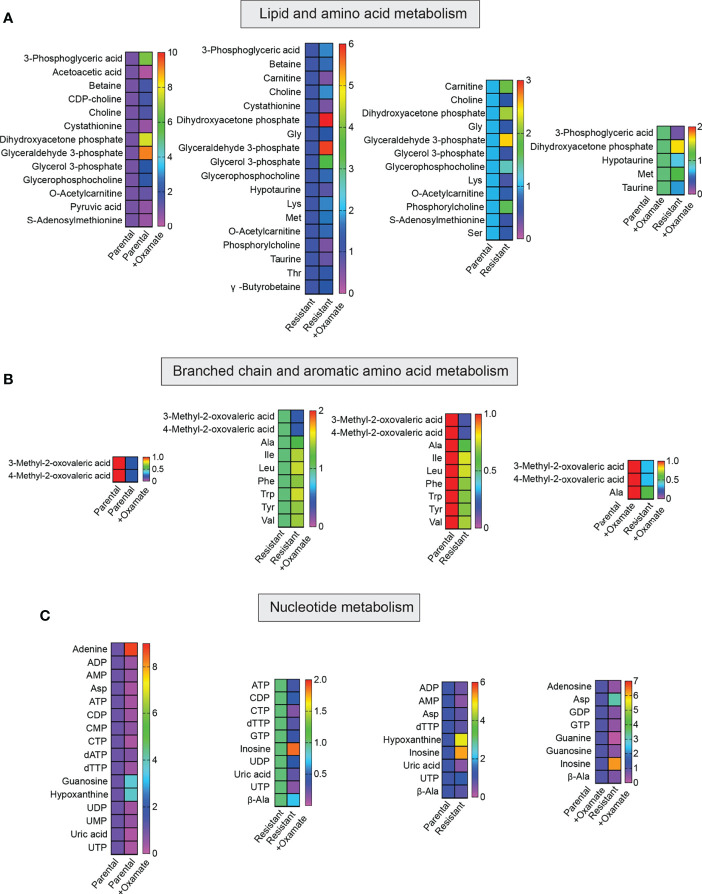
Metabolites generated during lipid, amino acid, and nucleotide metabolism in parental and oxamate-resistant cells treated with or without sodium oxamate. **(A–C)** Changes in the indicated metabolites generated during lipid, amino acid, and nucleotide metabolism under the indicated conditions.

**Figure 7:**
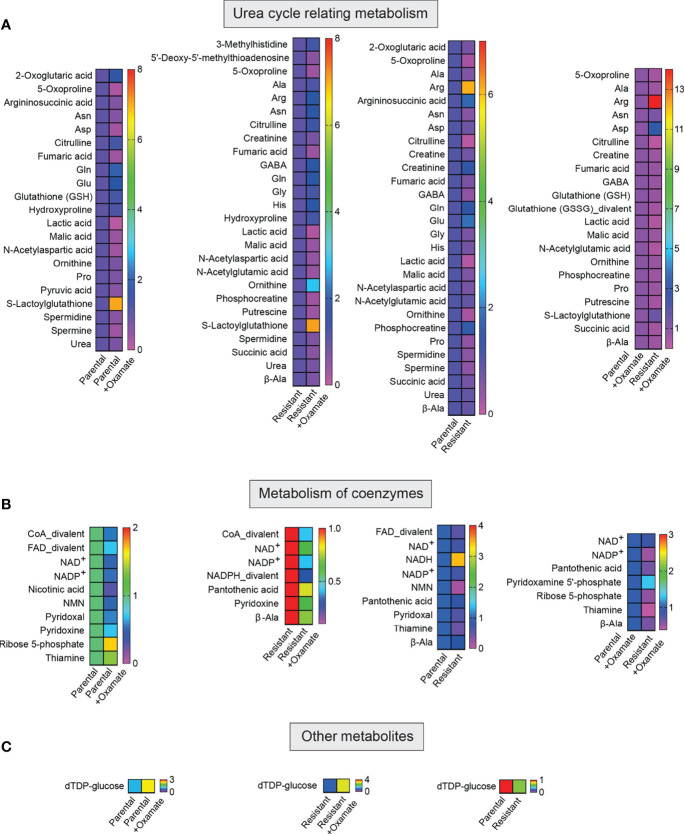
Metabolites associated with the metabolism of urea and coenzymes and other metabolites in parental and oxamate-resistant cells treated with or without sodium oxamate. **(A–C)** Changes in the indicated metabolites generated during the metabolism of urea and coenzymes and other metabolites under the indicated conditions.

We then performed untargeted metabolomics analyses in parental and oxamate-resistant MIAPaCa2 cells treated with and without oxamate using capillary electrophoresis time-of-flight mass spectrometry (CE-TOFMS), which detected a total of 228 different metabolites (**Figure 4B** and **Table S8**). As expected, the metabolomics analysis identified significant differences in lactic acid levels, which were significantly reduced in oxamate-treated parental MIAPaCa2 cells compared with untreated parental cells. Oxamate-resistant MIAPaCa2 cells also showed significantly reduced lactic acid levels compared with parental cells (**Figure 4C**). Furthermore, significant differences in the levels of other glycolytic pathway metabolites (**Figures 4D**, **E**) and those associated with other metabolic pathways were also observed (**Figures 5**–**7**). These results indicate that, similar to the differences observed in mRNA and chromatin accessibility profiles, metabolic profiles reveal major differences between parental and oxamate-resistant MIAPaCa2 cells and their responses to oxamate treatment.

### Alterations in the mRNA expression levels of metabolic genes in parental and oxamate-resistant PDAC cells and their enrichment in various metabolic pathways

**Figure 8:**
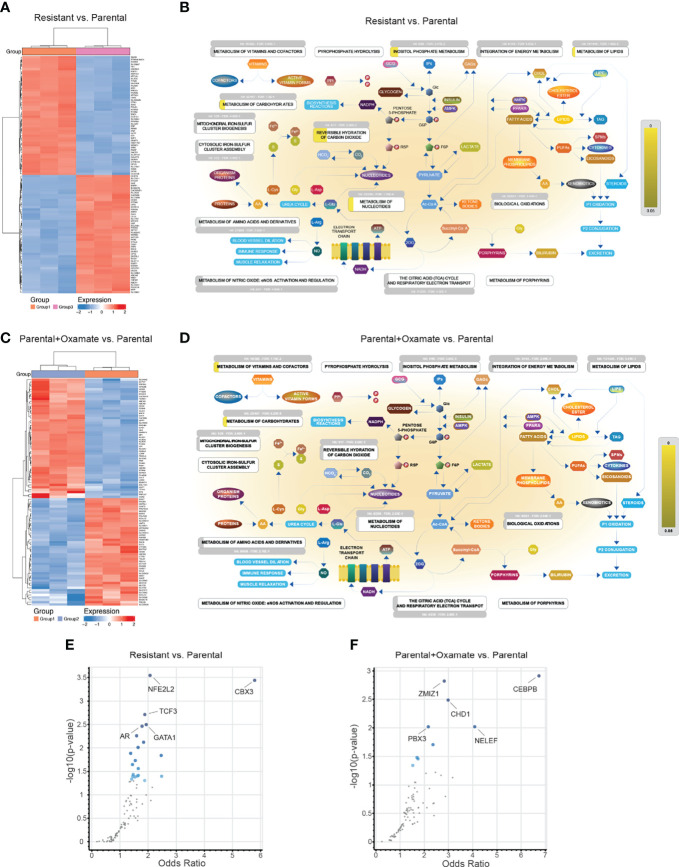
Alternations in metabolic genes and their potential transcriptional regulators in parental/resistant cells and parental/oxamate-treated parental cells. **(A)** Heatmap showing the expression of the top 100 (50 upregulated and 50 downregulated) differentially expressed metabolic genes between parental and oxamate-resistant MIAPaCa2 cells. **(B)** Reactome Pathway Analysis for differentially expressed metabolic genes between parental and oxamate-resistant MIAPaCa2 cells shows key metabolic pathways associated with significantly enriched genes (p-value <0.05, shown in yellow). **(C)** Heatmap showing the expression of the top 100 (50 upregulated and 50 downregulated) differentially expressed metabolic genes between vehicle- and oxamate-treated parental MIAPaCa2 cells. **(D)** Reactome Pathway Analysis for differentially expressed metabolic genes between vehicle- and oxamate-treated parental MIAPaCa2 cells shows key metabolic pathways associated with significantly enriched genes (p-value <0.05, shown in yellow). **(E)** Enrichr analysis using ENCODE and ChEA for identifying potential regulatory transcription factors involved in the differential expression of metabolic genes between parental and oxamate-resistant cells. **(F)** Enrichr analysis using ENCODE and ChEA for identifying potential regulatory transcription factors involved in the differential expression of metabolic genes between vehicle- and oxamate-treated parental MIAPaCa2 cells.

We next used our RNA-seq data to specifically examine the expression of metabolic genes and transporters using a combination of metabolic genes defined by the Kyoto Encyclopedia of Genes and Genomes (KEGG) and a list described in a previously published study that includes transporters able to impact metabolic pathways (**Table S9**) (30). The comparison between parental and oxamate-resistant MIAPaCa2 cells revealed the differential expression of 339 metabolic genes or transporters (**Table S10** and **Figure 8A**). Reactome Pathway Analysis of these 339 metabolic genes or transporters revealed that these differentially expressed genes were significantly enriched in pathways associated with the metabolism of lipids, carbohydrates, nucleotides, and inositol phosphate and the reversible hydration of carbon dioxides, among others (**Figure 8B** and **Table S11**). Similarly, the comparison between vehicle-treated and oxamate-treated parental MIAPaCa2 cells revealed 115 metabolic genes or transporters that were differentially expressed between these two conditions (**Figure 8C** and **Table S12**). Reactome Pathway Analysis of these 115 metabolic genes or transporters revealed that these differentially expressed genes were significantly enriched in pathways associated with the metabolism of carbohydrates, vitamins, and cofactors, among others (**Figure 8D** and **Table S13**).

Next, we attempted to identify potential transcription factors that regulate differentially expressed metabolic genes. We performed Enrichr analysis to identify differentially expressed metabolic genes in the comparison between parental and oxamate-resistant MIAPaCa2 cells and in the comparison between vehicle- and oxamate-treated parental MIAPaCa2 cells. In the comparison between parental and oxamate-resistant MIAPaCa2 cells, NFE2-like BZIP transcription factor 2 (NFE2L2), chromobox 3 (CBX3), TCF3, AR, and GATA-binding protein 1 (GATA1) were identified as the top-most significantly enriched transcription factors (**Figure 8E** and **Table S14**). In the comparison between parental and oxamate-treated MIAPaCa2 cells, CEBPB, zinc finger MIZ-type containing 1 (ZMIZ1), chromodomain helicase DNA–binding protein 1 (CHD1), negative elongation factor (NELF), and PBX3 were identified as the top-most significantly enriched transcription factors (**Figure 8F** and **Table S15**).

**Figure 9:**
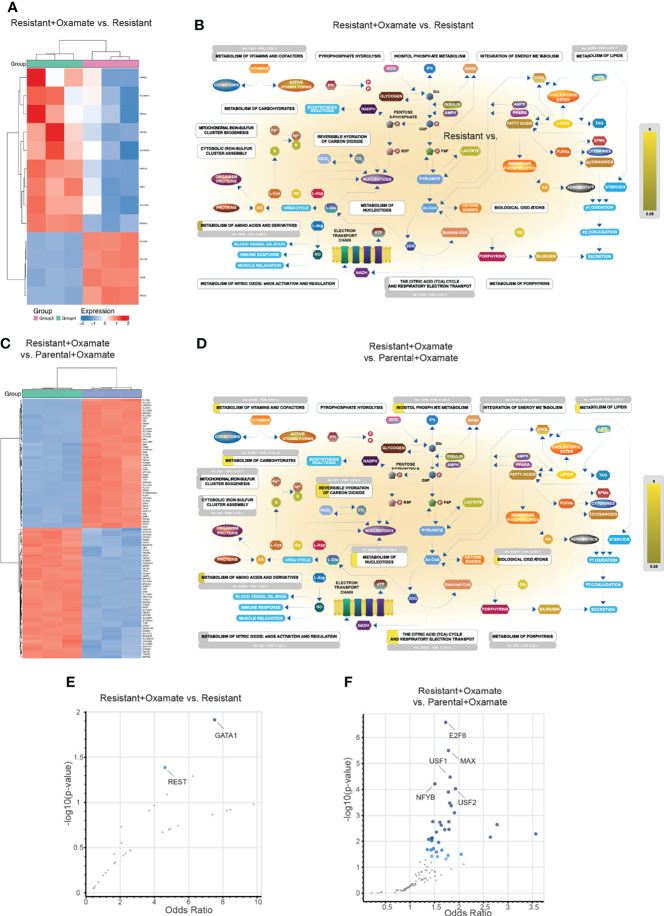
Alterations in metabolic genes and their potential transcriptional regulators in resistant/oxamate-treated resistant cells and oxamate-treated parental/oxamate-treated resistant cells. **(A)** Heatmap showing the expression of the top 100 (50 upregulated and 50 downregulated) differentially expressed metabolic genes between vehicle- and oxamate-treated oxamate-resistant MIAPaCa2 cells. **(B)** Reactome Pathway Analysis for differentially expressed metabolic genes between vehicle- and oxamate-treated oxamate-resistant MIAPaCa2 cells shows key metabolic pathways associated with significantly enriched genes (p-value <0.05, shown in yellow). **(C)** Heatmap showing the expression of the top 100 (50 upregulated and 50 downregulated) differentially expressed metabolic genes between oxamate-treated parental and oxamate-treated oxamate-resistant MIAPaCa2 cells. **(D)** Reactome Pathway Analysis for differentially expressed metabolic genes between oxamate-treated parental and oxamate-treated oxamate-resistant MIAPaCa2 cells cells shows key metabolic pathways associated with significantly enriched genes (p-value <0.05, shown in yellow). **(E)** Enrichr analysis using ENCODE and ChEA for identifying potential regulatory transcription factors involved in the differential expression of metabolic genes between vehicle- and oxamate-treated oxamate-resistant cells. **(F)** Enrichr analysis using ENCODE and ChEA for identifying potential regulatory transcription factors involved in the differential expression of metabolic genes between oxamate-treated parental and oxamate-treated oxamate-resistant MIAPaCa2 cells.

In the comparison between vehicle- and oxamate-treated oxamate-resistant MIAPaCa2 cells, only 13 genes were identified as differentially expressed (**Figure 9A** and **Table S16**); although these genes were not enriched in any major metabolic pathways (**Figure 9B**), some of these genes were enrichment in unconventional metabolic pathways, such as cellular hexose transport and vitamin C (ascorbate) metabolism (**Table S17**). Finally, in the comparison between oxamate-treated parental and oxamate-treated oxamate-resistant MIAPaCa2 cells, 540 differentially expressed metabolic genes or transporters were identified (**Figure 9C** and **Table S18**). Reactome Pathway Analysis of these 540 metabolic genes or transporters revealed the significant enrichment of pathways associated with the metabolism of carbohydrates, vitamins, and cofactors, among others (**Figure 9D** and **Table S19**).

Next, we identified transcription factors potentially involved in the regulation of differentially expressed metabolic genes. To do so, we performed an Enrichr analysis of the differentially expressed metabolic genes in the comparisons between vehicle- and oxamate-treated oxamate-resistant MIAPaCa2 cells and between oxamate-treated parental and oxamate-treated oxamate-resistant MIAPaCa2 cells. In the comparison between vehicle- and oxamate-treated oxamate-resistant MIAPaCa2 cells, GATA1 and repressor element 1–silencing transcription factor (REST) were identified as the top-most significantly enriched transcription factors (**Figure 9E** and **Table S20**). In the comparison between oxamate-treated parental and oxamate-resistant MIAPaCa2 cells, E2F transcription factor 6 (E2F6), Myc-associated factor X (MAX), upstream transcription factor (USF)1, USF2, and nuclear transcription factor Y subunit beta (NFYB) were identified as the top-most significantly enriched transcription factors (**Figure 9F** and **Table S21**).

### Integration of metabolic and RNA-seq data to identity co-regulated pathways at both the transcriptional and metabolic levels

**Figure 10:**
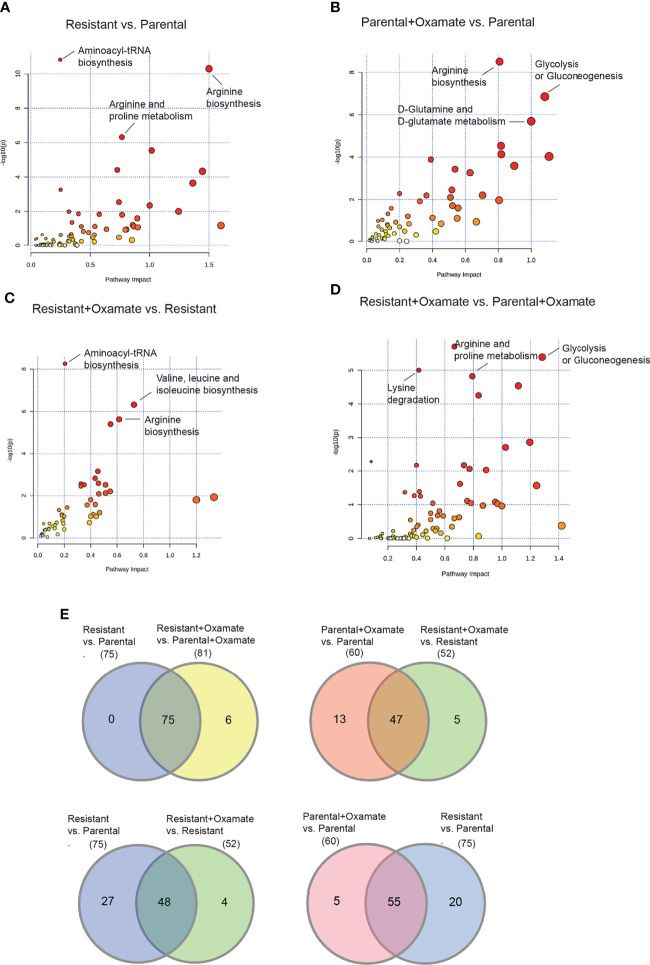
Integrative RNA-seq and metabolomics analysis identifies similarities and differences between parental and oxamate-resistant pancreatic cancer cells. **(A)** Integration of altered metabolic genes and metabolites was performed using the joint pathway analysis module of MetaboAnalyst 5.0 for the comparison between parental and oxamate-resistant MIAPaCa2 cells. **(B)** Integration of altered metabolic genes and metabolites was performed using the joint pathway analysis module of MetaboAnalyst 5.0. for the comparison between vehicle- and oxamate-treated parental MIAPaCa2 cells. **(C)** Integration of altered metabolic genes and metabolites was performed using the joint pathway analysis module of MetaboAnalyst 5.0 for the comparison between vehicle- and oxamate-treated oxamate-resistant MIAPaCa2 cells. **(D)** Integration of altered metabolic genes and metabolites was performed using the joint pathway analysis module of MetaboAnalyst 5.0 for the comparison between oxamate-treated parental and oxamate-treated oxamate-resistant MIAPaCa2 cells. **(E)** Venn diagram for the indicated comparisons showing differentially altered metabolic pathways based on the integrative analysis of differentially expressed metabolic genes identified by RNA-seq and differential metabolites identified by the metabolomics analysis in the indicated samples.

To identify metabolic pathways and mRNA expression levels that are co-regulated, we performed an integrative analysis combining RNA-seq and metabolomics data. We focused on all metabolic genes identified as altered across the various comparisons (parental versus oxamate-resistant; vehicle- versus oxamate-treated parental; vehicle- versus oxamate-treated oxamate-resistant; and oxamate-treated parental versus oxamate-resistant). To conduct the integrated analysis, we used the joint pathway analysis tool in MetaboAnalyst 5.0 (31). The results of this integrated analysis are shown in **Figures 10A**–**D** and summarized in **Tables S22**–**S25**. For example, aminoacyl tRNA biosynthesis, arginine biosynthesis, and proline biosynthesis were identified as the three top-most significantly altered metabolic pathways in the integrated analysis for parental versus oxamate-resistant MIAPaCa2 cells. We also analyzed differences among comparisons to identify specific changes associated with specific pairwise comparisons. The Venn diagram for this analysis is shown in **Figure 10E**. For example, 75 metabolic pathways were identified as significantly altered in the integrated analysis for parental versus oxamate-resistant MIAPaCa2 cells, whereas 81 metabolic pathways were identified as significantly altered in oxamate-treated parental versus oxamate-resistant MIAPaCa2 cells. Of these, 75 were common between the two comparisons, 0 were specific to the comparison between parental and oxamate-resistant MIAPaCa2 cells, and 6 were specifically altered in the comparison between oxamate-treated parental and oxamate-resistant MIAPaCa2 cells. These results demonstrate that the integration of metabolomics and RNA-seq data can reveal information that is not possible to identify by looking at either RNA-seq or metabolomics data alone.

## Discussion

Targeting metabolic enzymes has emerged as a new approach for cancer therapy (3, 32). However, based on our knowledge of the response of cancer cells to other therapeutic agents, including the development of eventual therapeutic resistance, cancer cells are expected to eventually adapt to metabolic enzyme inhibition. Therefore, understanding the adaptation process at the molecular level is necessary to optimize the use of metabolic enzyme–targeting agents and improve the treatment or prevent the development of drug-resistant cancers.

The overexpression of the metabolic enzyme LDHA is necessary for tumor growth and progression (6–10, 13–15). LDHA typically exists as a tetramer (LDH-5) and functions primarily to interconvert pyruvate to lactate and transform NADH to NAD+. However, LDHA is a multifunctional protein and has been localized not only to the cytoplasm but also in mitochondria and the nucleus (33, 34). Outside of the nucleus, LDHA plays a role in the regulation of glycolysis, whereas LDHA inside the nucleus functions as a single-stranded DNA–binding protein (SSB) (34). Additionally, nuclear LDHA has been shown to sense reactive oxygen species, leading to the production of alpha-hydroxybutyrate in human papillomavirus (HPV)-induced cervical cancer (35).

Here, using the LDHAi oxamate as a metabolic enzyme inhibitory drug and PDAC as a model system, we studied the impact of LDHA inhibition on the transcriptional, chromatin, and metabolomics landscapes. We also performed integrated analyses to identify common transcriptome and chromatin accessibility features and common transcriptome and metabolic features in parental and oxamate-resistant PDAC cells treated with and without oxamate. These studies revealed several differences in the mRNA expression profiles, chromatin accessibility profiles, and metabolic profiles across parental and oxamate-resistant PDAC cells treated with or without oxamate.

RNA-seq analysis showed that parental cells were highly responsive to oxamate treatment, whereas oxamate-resistant cells showed only marginal responses to oxamate treatment. Consistent with this finding, vehicle- and oxamate-treated parental cells displayed large differences in their mRNA expression profiles. These results are consistent with the rationale that once oxamate resistance develops, oxamate-resistant cells become largely inert in response to oxamate treatment. In conclusion, these results demonstrate that oxamate resistant cells may acquire such a state as a mechanism of adaptation to survive in the presence of oxamate and to become oxamate resistant. Notably, also pathway analysis identified distinct set of genes and pathways gets enriched in each of treatment conditions, in which oxamate treated parental cells very clearly separated from highly non-responsive oxamate resistant cells.

Furthermore, ATAC-seq analysis and its integration with the RNA-seq analysis revealed that only a small subset of mRNAs (1% to 12%) associated with differential expression were associated with changes in chromatin accessibility within promoter regions. These results are consistent with those of other studies, which also reported an approximately 10% overlap between RNA-seq and ATAC-seq data (36). Furthermore, the usefulness of integrated analyses was highlighted by the ability of integrated RNA-seq and ATAC-seq data to identify changes in chromatin accessibility associated with consequent changes in mRNA expression, which allowed for the identification of potential transcriptional regulators associated with changes in mRNA expression. Additionally, using the integration data with that of transcriptional regulator identification data allows for identifying distinct transcription factors that might contribute to distinct transcriptional response to oxamate treatment. Future studies will allow us and others to evaluate the role of these individual transcription factors in driving the oxamate resistant state and their role in glucose metabolism regulation in part via glycolysis regulation.

Finally, the integration of metabolomics data with RNA-seq data for metabolic genes and transporters allowed changes in mRNA levels to be linked with changes in metabolites. These studies thereby provide an overall reason for changes in metabolites under distinct oxamate treatment or parental and oxamate-resistant states, and demonstrate that they in part are driven due to changes at transcriptional of enzymes that in turn regulate various metabolic pathways.

In conclusion, these studies highlight the importance of performing integrative analyses of RNA-seq, ATAC-seq, and metabolomics data to identify changes that would not be possible to identify using any single omics–based approach. Further studies using other functional genomics approaches are likely to reveal the driving roles played by these transcriptional and metabolic changes in conferring resistance to LDHAis. Similar approaches can be employed to identify differences in the response to other metabolic inhibitors, which may eventually lead to the optimization of metabolic enzyme–targeted therapies to achieve the maximum clinical benefits in cancer patients.

Future studies using a larger number of PDAC cell lines, in vivo models, and other cancer types of glycolytic nature will allow us to further identifying unifying features of oxamate resistant cells. Similarly, use of other LDHA inhibitors and comparing the genetic inhibition of LDHA with that or its pharmacological inhibition will reveal the impact of non-enzymatic and non-canonical functions of LDHA in promoting growth of cancer cells.

## Materials and methods

### Cell culture

MIAPaCa2 cells were obtained from the American Type Culture Collection (Manassas, VA, USA) and were grown in Dulbecco’s modified Eagle medium (Life Technologies, ThermoFisher Scientific, Waltham, MA, USA), supplemented with 10% fetal bovine serum (Life Technologies, ThermoFisher Scientific) and 1% penicillin/streptomycin (Life Technologies), at a CO2 concentration of 5%.

### Generation of sodium oxamate–resistant cell lines

Sodium oxamate–resistant cells were generated by continuously culturing MIAPaCa2 cells in the presence of 10 mM sodium oxamate for 4 weeks. The medium was replaced every 3 days and supplemented with fresh sodium oxamate until these cells began to form distinct colonies. The sodium oxamate–resistant phenotype in isolated polyclonal populations was further validated by clonogenic assay.

### Clonogenic assays

The clonogenic ability of MIAPaCA2 parental and sodium oxamate–resistant cells was measured under untreated and sodium oxamate–treated conditions. For these assays, 5 × 10^3^ cells/well were seeded in 6-well culture plates. For drug treatment experiments, cells were treated with 10 mM of sodium oxamate. The medium containing sodium oxamate was changed every 3 days. After 10–14 days of treatment, surviving colonies were stained with a solution containing 40% methanol, 10% acetic acid, and 0.005% Coomassie Brilliant Blue R-250 (Sigma-Aldrich, USA), and the plates were imaged using an Epson Perfection V850 Pro Photo Scanner (USA).

### Seahorse analysis for the measurement of extracellular acidification rates

Extracellular acidification rates were measured using the XFe24 analyzer (Seahorse Bioscience). Briefly, parental and oxamate-resistant MIAPaCa2 cells (untreated and treated with sodium oxamate) were seeded at a density of 40,000 cells/well in triplicate. After 24 h, the culture medium was replaced with assay medium, and extracellular acidification rates were measured for 2 h.

### RNA-sequencing

Total RNA was extracted from frozen cell pellet samples using the Qiagen RNeasy Plus Universal mini kit according to the manufacturer’s instructions (Qiagen, Hilden, Germany). RNA samples were quantified using a Qubit 2.0 Fluorometer (Life Technologies, Carlsbad, CA, USA), and RNA integrity was verified using an Agilent TapeStation 4200 (Agilent Technologies, Palo Alto, CA, USA). RNA-seq libraries were prepared using the NEBNext Ultra RNA Library Prep Kit for Illumina according to the manufacturer’s instructions (NEB, Ipswich, MA, USA). Briefly, mRNAs were initially enriched with Oligo(dT) beads. Enriched mRNAs were fragmented for 15 minutes at 94°C. First-strand and second-strand cDNA were subsequently synthesized. cDNA fragments were end-repaired and adenylated at the 3’ends, and universal adapters were ligated to cDNA fragments, followed by index addition and library enrichment by PCR with limited cycles. The sequencing library was validated on the Agilent TapeStation (Agilent Technologies, Palo Alto, CA, USA) and quantified using a Qubit 2.0 Fluorometer (Invitrogen, Carlsbad, CA) and by quantitative PCR (KAPA Biosystems, Wilmington, MA, USA).

The sequencing libraries were clustered on two lanes of a flowcell. After clustering, the flowcell was loaded on the Illumina HiSeq instrument (4000 or equivalent) according to the manufacturer’s instructions. The samples were sequenced using a 2 × 150-bp paired-end configuration. Image analysis and base calling were conducted using HiSeq Control Software. Raw sequence data (.bcl files) generated from Illumina HiSeq were converted into fastq files and de-multiplexed using Illumina bcl2fastq 2.17 software. One mismatch was allowed for index sequence identification.

### RNA-sequencing analysis

After investigating the quality of the raw data, sequence reads were trimmed to remove possible adapter sequences and nucleotides with poor quality using Trimmomatic v.0.36. The trimmed reads were mapped to the reference genome available on ENSEMBL using the STAR aligner v.2.5.2b. The STAR aligner uses a splice aligner that detects splice junctions and incorporates them to help align the entire read sequences. BAM files were generated during this step. Unique gene hit counts were calculated by using feature counts from the Subread package v.1.5.2. Only unique reads that fell within exon regions were counted.

The reads were first mapped to the latest UCSC transcript set using Bowtie2 version 2.1.0 (37), and the gene expression level was estimated using RSEM v1.2.15 (38). Differentially expressed genes were identified using the DESeq2 program (39). Genes showing altered expression with p < 0.05 and fold changes > 1.5 were considered to be differentially expressed. Goseq (40) was used to perform the gene ontology (GO) enrichment analysis, and Kobas was used to perform the pathway analysis (41).

### ATAC-sequencing and data analysis

Parental or oxamate-resistant MIAPaCa2 cells treated with 10 mM oxamate or phosphate-buffered saline (PBS) for 24 h were washed and treated with DNAse I (Life Tech, Cat. #EN0521) to remove genomic DNA contamination. Live cell samples were quantified and assessed for viability using a Countess Automated Cell Counter (ThermoFisher Scientific, Waltham, MA, USA). After cell lysis and cytosol removal, nuclei were treated with Tn5 enzyme (Illumina, Cat. #20034197) for 30 min at 37°C and purified with a MinElute PCR Purification Kit (Qiagen, Cat. #28004) to produce tagmented DNA samples. Tagmented DNA was barcoded with a Nextera Index Kit v2 (Illumina, Cat. #FC-131-2001) and amplified via PCR prior to an SPRI Bead cleanup to yield purified DNA libraries.

The reads were first mapped to the latest UCSC genome set using Bowtie2 version 2.1.0 (37). Mitochondrial reads, duplicate reads, and non-unique reads were removed before peak calling. MACS2 was used for peak calling using BAMPE mode (42). Differentially expressed peaks were identified using the DEseq2 program (39). Peaks showing altered expression with p < 0.05 and fold change > 1.5 were considered differentially expressed. Downstream genes of the differential peaks were used for GO and pathway enrichment analysis. Goseq (40) was used to perform the GO enrichment analysis, and Kobas was used to perform the pathway analysis (41).

### Integrated analysis of RNA-seq and ATAC-seq data

RNA-seq and ATAC-seq data were analyzed to identify same-direction changes in mRNA expression and chromatin accessibility. This integration was used to assess pathway enrichment using Reactome Pathway Analysis.

### Enrichment analysis for transcription factor binding sites

Transcription factor enrichment analysis was performed by Enrichr, a web-based enrichment analysis using ENCODE and the ChEA consensus TFs from the ChIP-X option for identifying potential regulatory transcription factors involved in the differential expression of metabolic genes between parental and oxamate-resistant MIAPaCa2 cells treated with and without sodium oxamate. The gene set of differentially expressed genes was used as input. To identify transcription factors using data from multiple experiments, set intersection was applied to obtain consensus. For each comparison, multiple transcription factors were identified as potentially direct transcriptional regulators for differentially expressed mRNAs.

### Reactome pathway analysis

The differentially expressed genes identified in the RNA-seq or ATAC-seq analyses of metabolites identified by metabolomics analysis were evaluated using Reactome Pathway Analysis using the Reactome Pathway Analysis tool version 80 (https://www.reactome.org). Briefly, common differentially expressed genes among parental and oxamate-resistant MIAPaCa2 cells treated with and without sodium oxamate were uploaded into the Reactome Pathway Analysis tool. In this analysis, all non-human identifiers were converted to their human equivalents. The 25 most significantly altered pathways specific to Homo sapiens were identified using all resources and sorted by p-values.

### Metabolomics analysis

Parental and oxamate-resistant MIAPaCa2 cells treated with and without oxamate were analyzed for alterations in metabolic pathways using the CE-TOFMS–based scan profiling method developed by Human Metabolome Technologies (Boston, MA, USA) using an Agilent CE-TOFMS system (Agilent Technologies Inc.). Parental and oxamate-resistant cells were grown in the absence and presence of 10 mM sodium oxamate in triplicate, and 1 × 106 cells were analyzed for each condition. Samples were prepared according to the recommendations of Human Metabolome Technologies. The samples were mixed with 800 µL methanol, followed by the addition of 450 µL Milli-Q water containing internal standards (10 µM), and mixed thoroughly. The extract (1,000 µL) was centrifuged (2,300 × g, 4°C, 5 min). The supernatant (350 µL) was filtrated through a 5-kDa cutoff filter (ULTRAFREE-MC-PLHCC, Human Metabolome Technologies, Yamagata, Japan) to remove macromolecules. The filtrate was concentrated by centrifugation and resuspended in 50 µL ultrapure water immediately before the measurement. Metabolome analysis was performed in samples of cultured cells using CE-TOFMS for cationic and anionic metabolites. For data analysis, peaks detected during spectrometric analysis were extracted using the MasterHands version 2.17.1.11 automated integration software (developed at Keio University, Tokyo, Japan) to determine the mass/charge ratio (m/z), migration time, and peak area. Peak area was converted to relative peak area using the following equation: Relative peak area = Metabolite Peak Area/Internal Standard Peak Area × Sample Amount. The peak detection limit was determined based on a signal-to-noise ratio of 3. Putative metabolites were assigned based on the m/z value and migration time using Human Metabolomic Technologies’ standard library and known–unknown peak library. The tolerance was ±0.5 min for migration time and ±10 ppm for m/z. Hierarchical cluster analysis and principal component analysis were performed using statistical analysis software (developed at Human Metabolome Technologies). In total, 228 metabolites were detected (121 metabolites in cation mode and 107 metabolites in anion mode) and annotated using Human Metabolomic Technologies’ standard library and known–unknown peak library. All metabolite concentrations were calculated by normalizing the peak area of each metabolite to the area of the internal standard and by comparing with standard curves obtained from a 100 µM single-point calibration. The peak profiles of putative metabolites were represented on metabolic pathway maps using the Visualization and Analysis of Networks containing Experimental Data (VANTED) software (http://vanted.ipk-gatersleben.de/). The pathway map was prepared based on metabolic pathways known to exist in human cells according to information in the KEGG database (http://www.genome.jp/kegg/). The list of fold changes for altered metabolites identified in each comparison is shown in **Table S8**.

### Integrated analysis for RNA-seq and metabolomics data

Differential genes and metabolites with fold changes were combined and used as the input file in the MetaboAnalyst 5.0 joint pathway analysis tool. MetaboAnalyst supports raw MS spectra processing, comprehensive data normalization, statistical analysis, functional analysis, meta-analysis, and integrative analysis with other omics data. The most significantly enriched pathways were selected and plotted.

## Data availability statement

The RNA-seq data and ATAC-seq data was submitted to Gene Expresison Omnibus. (GEO). The accession number for the RNA-seq data is GSE164976 and the accession number for ATAC-seq data is GSE164974. Metabolomics data and other relevant spread sheets can be found in the article/**Supplementary material**.

## Author contributions

NW and RG conceived the project. PM, VR, RG, and NW designed the experiments. PM and VR performed the research. PM, VR, RG, and NW analyzed and interpreted the data. PM, RG, and NW wrote the paper. All authors reviewed, made suggestions, approved the paper, and provided comments.

## Acknowledgments

We gratefully acknowledge grants from the National Institutes of Health (NIH) [R01CA195077 (to NW), R01CA200919 (to NW), 1R01CA218008 (to NW), and R01CA233481 (to RG)].

## Conflict of interest

The authors declare that the research was conducted in the absence of any commercial or financial relationships that could be construed as a potential conflict of interest.

## Publisher’s note

All claims expressed in this article are solely those of the authors and do not necessarily represent those of their affiliated organizations, or those of the publisher, the editors and the reviewers. Any product that may be evaluated in this article, or claim that may be made by its manufacturer, is not guaranteed or endorsed by the publisher.

## References

[1] Martinez-Reyes I Chandel NS. Cancer metabolism: Looking forward. Nat Rev Cancer (2021) 21(10):669–80. doi: 10.1038/s41568-021-00378-6 34272515

[2] Nagarajan A Malvi P Wajapeyee N. Oncogene-directed alterations in cancer cell metabolism. Trends Cancer (2016) 2(7):365–77. doi: 10.1016/j.trecan.2016.06.002 PMC5096652 27822561

[3] Stine ZE Schug ZT Salvino JM Dang CV. Targeting cancer metabolism in the era of precision oncology. Nat Rev Drug Discovery (2022) 21(2):141–62. doi: 10.1038/s41573-021-00339-6 PMC8641543 34862480

[4] Sreedhar A Zhao Y. Dysregulated metabolic enzymes and metabolic reprogramming in cancer cells. BioMed Rep (2018) 8(1):3–10. doi: 10.3892/br.2017.1022 29399334 PMC5772474

[5] Nilsson R Jain M Madhusudhan N Sheppard NG Strittmatter L Kampf C. Metabolic enzyme expression highlights a key role for Mthfd2 and the mitochondrial folate pathway in cancer. Nat Commun (2014) 5:3128. doi: 10.1038/ncomms4128 24451681 PMC4106362

[6] Yang Y Chong Y Chen M Dai W Zhou X Ji Y. Targeting lactate dehydrogenase a improves radiotherapy efficacy in non-small cell lung cancer: From bedside to bench. J Transl Med (2021) 19(1):170. doi: 10.1186/s12967-021-02825-2 33902615 PMC8074241

[7] An J Zhang Y He J Zang Z Zhou Z Pei X. Lactate dehydrogenase a promotes the invasion and proliferation of pituitary adenoma. Sci Rep (2017) 7(1):4734. doi: 10.1038/s41598-017-04366-5 28680051 PMC5498590

[8] Yu Y Liao M Liu R Chen J Feng H Fu Z. Overexpression of lactate dehydrogenase-a in human intrahepatic cholangiocarcinoma: Its implication for treatment. World J Surg Oncol (2014) 12:78. doi: 10.1186/1477-7819-12-78 24679073 PMC4230420

[9] Zhao J Huang X Xu Z Dai J He H Zhu Y. Ldha promotes tumor metastasis by facilitating epithelialmesenchymal transition in renal cell carcinoma. Mol Med Rep (2017) 16(6):8335–44. doi: 10.3892/mmr.2017.7637 28983605

[10] Wu J You K Chen C Zhong H Jiang Y Mo H. High pretreatment ldh predicts poor prognosis in hypopharyngeal cancer. Front Oncol (2021) 11:641682. doi: 10.3389/fonc.2021.641682 33777804 PMC7991725

[11] Valvona CJ Fillmore HL Nunn PB Pilkington GJ. The regulation and function of lactate dehydrogenase a: Therapeutic potential in brain tumor. Brain Pathol (2016) 26(1):3–17. doi: 10.1111/bpa.12299 26269128 PMC8029296

[12] Feng Y Xiong Y Qiao T Li X Jia L Han Y. Lactate dehydrogenase a: A key player in carcinogenesis and potential target in cancer therapy. Cancer Med (2018) 7(12):6124–36. doi: 10.1002/cam4.1820 PMC6308051 30403008

[13] Fantin VR St-Pierre J Leder P. Attenuation of ldh-a expression uncovers a link between glycolysis, mitochondrial physiology, and tumor maintenance. Cancer Cell (2006) 9(6):425–34. doi: 10.1016/j.ccr.2006.04.023 16766262

[14] Qing G Skuli N Mayes PA Pawel B Martinez D Maris JM. Combinatorial regulation of neuroblastoma tumor progression by n-myc and hypoxia inducible factor hif-1alpha. Cancer Res (2010) 70(24):10351–61. doi: 10.1158/0008-5472.CAN-10-0740 PMC3005134 20961996

[15] Xie H Hanai J Ren JG Kats L Burgess K Bhargava P. Targeting lactate dehydrogenase–a inhibits tumorigenesis and tumor progression in mouse models of lung cancer and impacts tumor-initiating cells. Cell Metab (2014) 19(5):795–809. doi: 10.1016/j.cmet.2014.03.003 24726384 PMC4096909

[16] Billiard J Dennison JB Briand J Annan RS Chai D Colon M. Quinoline 3-sulfonamides inhibit lactate dehydrogenase a and reverse aerobic glycolysis in cancer cells. Cancer Metab (2013) 1(1):19. doi: 10.1186/2049-3002-1-19 24280423 PMC4178217

[17] Oshima N Ishida R Kishimoto S Beebe K Brender JR Yamamoto K. Dynamic imaging of ldh inhibition in tumors reveals rapid in vivo metabolic rewiring and vulnerability to combination therapy. Cell Rep (2020) 30(6):1798–810.e4. doi: 10.1016/j.celrep.2020.01.039 32049011 PMC7039685

[18] Boudreau A Purkey HE Hitz A Robarge K Peterson D Labadie S. Metabolic plasticity underpins innate and acquired resistance to ldha inhibition. Nat Chem Biol (2016) 12(10):779–86. doi: 10.1038/nchembio.2143 27479743

[19] Qiao T Xiong Y Feng Y Guo W Zhou Y Zhao J. Inhibition of ldh-a by oxamate enhances the efficacy of anti-Pd-1 treatment in an nsclc humanized mouse model. Front Oncol (2021) 11:632364. doi: 10.3389/fonc.2021.632364 33859941 PMC8042335

[20] Altinoz MA Ozpinar A. Oxamate targeting aggressive cancers with special emphasis to brain tumors. BioMed Pharmacother (2022) 147:112686. doi: 10.1016/j.biopha.2022.112686 35124385

[21] Swinnen JV Brusselmans K Verhoeven G. Increased lipogenesis in cancer cells: New players, novel targets. Curr Opin Clin Nutr Metab Care (2006) 9(4):358–65. doi: 10.1097/01.mco.0000232894.28674.30 16778563

[22] Costello LC Franklin RB. 'Why do tumour cells glycolyse?': From glycolysis through citrate to lipogenesis. Mol Cell Biochem (2005) 280(1-2):1–8. doi: 10.1007/s11010-005-8841-8 16511951 PMC4461431

[23] Daemen A Peterson D Sahu N McCord R Du X Liu B. Metabolite profiling stratifies pancreatic ductal adenocarcinomas into subtypes with distinct sensitivities to metabolic inhibitors. Proc Natl Acad Sci U S A (2015) 112(32):E4410–7. doi: 10.1073/pnas.1501605112 PMC4538616 26216984

[24] Rong Y Wu W Ni X Kuang T Jin D Wang D. Lactate dehydrogenase a is overexpressed in pancreatic cancer and promotes the growth of pancreatic cancer cells. Tumour Biol (2013) 34(3):1523–30. doi: 10.1007/s13277-013-0679-1 23404405

[25] Mohammad GH Olde Damink SW Malago M Dhar DK Pereira SP. Pyruvate kinase M2 and lactate dehydrogenase a are overexpressed in pancreatic cancer and correlate with poor outcome. PLoS One (2016) 11(3):e0151635. doi: 10.1371/journal.pone.0151635 26989901 PMC4798246

[26] Le A Cooper CR Gouw AM Dinavahi R Maitra A Deck LM. Inhibition of lactate dehydrogenase a induces oxidative stress and inhibits tumor progression. Proc Natl Acad Sci U S A (2010) 107(5):2037–42. doi: 10.1073/pnas.0914433107 PMC2836706 20133848

[27] Cheng CS Tan HY Wang N Chen L Meng Z Chen Z. Functional inhibition of lactate dehydrogenase suppresses pancreatic adenocarcinoma progression. Clin Transl Med (2021) 11(6):e467. doi: 10.1002/ctm2.467 34185423 PMC8238920

[28] Li B Carey M Workman JL. The role of chromatin during transcription. Cell (2007) 128(4):707–19. doi: 10.1016/j.cell.2007.01.015 17320508

[29] Chen EY Tan CM Kou Y Duan Q Wang Z Meirelles GV. Enrichr: Interactive and collaborative Html5 gene list enrichment analysis tool. BMC Bioinf (2013) 14:128. doi: 10.1186/1471-2105-14-128 PMC3637064 23586463

[30] Possemato R Marks KM Shaul YD Pacold ME Kim D Birsoy K. Functional genomics reveal that the serine synthesis pathway is essential in breast cancer. Nature (2011) 476(7360):346–50. doi: 10.1038/nature10350 PMC3353325 21760589

[31] Pang Z Chong J Zhou G de Lima Morais DA Chang L Barrette M. Metaboanalyst 5.0: Narrowing the gap between raw spectra and functional insights. Nucleic Acids Res (2021) 49(W1):W388–W96. doi: 10.1093/nar/gkab382 PMC8265181 34019663

[32] Galluzzi L Kepp O Vander Heiden MG Kroemer G. Metabolic targets for cancer therapy. Nat Rev Drug Discovery (2013) 12(11):829–46. doi: 10.1038/nrd4145 24113830

[33] Cattaneo A Biocca S Corvaja N Calissano P. Nuclear localization of a lactic dehydrogenase with single-stranded DNA-binding properties. Exp Cell Res (1985) 161(1):130–40. doi: 10.1016/0014-4827(85)90497-5 3902489

[34] Brooks GA Dubouchaud H Brown M Sicurello JP Butz CE. Role of mitochondrial lactate dehydrogenase and lactate oxidation in the intracellular lactate shuttle. Proc Natl Acad Sci U S A (1999) 96(3):1129–34. doi: 10.1073/pnas.96.3.1129 PMC15362 9927705

[35] Liu Y Guo JZ Liu Y Wang K Ding W Wang H. Nuclear lactate dehydrogenase a senses ros to produce alpha-hydroxybutyrate for hpv-induced cervical tumor growth. Nat Commun (2018) 9(1):4429. doi: 10.1038/s41467-018-06841-7 30356100 PMC6200739

[36] Miao W Ma Z Tang Z Yu L Liu S Huang T. Integrative atac-seq and rna-seq analysis of the longissimus muscle of luchuan and duroc pigs. Front Nutr (2021) 8:742672. doi: 10.3389/fnut.2021.742672 34660666 PMC8511529

[37] Langmead B Salzberg SL. Fast gapped-read alignment with bowtie 2. Nat Methods (2012) 9(4):357–9. doi: 10.1038/nmeth.1923 PMC3322381 22388286

[38] Li B Dewey CN. Rsem: Accurate transcript quantification from rna-seq data with or without a reference genome. BMC Bioinf (2011) 12:323. doi: 10.1186/1471-2105-12-323 PMC3163565 21816040

[39] Love MI Huber W Anders S. Moderated estimation of fold change and dispersion for rna-seq data with Deseq2. Genome Biol (2014) 15(12):550. doi: 10.1186/s13059-014-0550-8 25516281 PMC4302049

[40] Young MD Wakefield MJ Smyth GK Oshlack A. Gene ontology analysis for rna-seq: Accounting for selection bias. Genome Biol (2010) 11(2):R14. doi: 10.1186/gb-2010-11-2-r14 20132535 PMC2872874

[41] Xie C Mao X Huang J Ding Y Wu J Dong S. Kobas 2.0: A web server for annotation and identification of enriched pathways and diseases. Nucleic Acids Res (2011) 39(Web Server issue):W316–22. doi: 10.1093/nar/gkr483 PMC3125809 21715386

[42] Zhang Y Liu T Meyer CA Eeckhoute J Johnson DS Bernstein BE. Model-based analysis of chip-seq (Macs). Genome Biol (2008) 9(9):R137. doi: 10.1186/gb-2008-9-9-r137 18798982 PMC2592715

