# Supplementary material for: Transcriptional, chromatin, and metabolic landscapes of LDHA inhibitor–resistant pancreatic ductal adenocarcinoma
Source: Front Oncol. 2022 Aug 2;12:926437. doi: 10.3389/fonc.2022.926437 (PMC9378957; doi:10.3389/fonc.2022.926437)
Supplement: Supplementary file 1 [file DataSheet_1.zip › Ziped tables/Table S19_Group 2 vs Group 4_Reactome analysis.docx]

**Table S19.** List of the top 25 most significantly altered metabolic pathways from the RNA-sequencing analysis performed in oxamate-treated parental (oxamate-sensitive) and oxamate-resistant MIAPaCa2 cells using the Reactome Pathway Analysis tool

| **Pathway Name** | **Entities** | | | | **Reactions** | |
| --- | --- | --- | --- | --- | --- | --- |
|  | **Found** | **Ratio** | **p-value** | **False Discovery Rate** | **Found** | **Ratio** |
| Metabolism of carbohydrates | 64 / 457 | 0.03 | 1.11e-16 | 5.91e-14 | 74 / 243 | 0.018 |
| Metabolism | 343 / 3,643 | 0.242 | 1.11e-16 | 5.91e-14 | 530 / 2,251 | 0.165 |
| Transport of small molecules | 87 / 966 | 0.064 | 3.18e-11 | 1.13e-08 | 102 / 443 | 0.032 |
| SLC-mediated transmembrane  transport | 48 / 421 | 0.028 | 1.05e-09 | 2.79e-07 | 55 / 191 | 0.014 |
| Metabolism of lipids | 109 / 1,446 | 0.096 | 2.33e-09 | 4.97e-07 | 179 / 955 | 0.07 |
| Glucose metabolism | 23 / 140 | 0.009 | 5.97e-08 | 1.06e-05 | 23 / 50 | 0.004 |
| Glycolysis | 20 / 110 | 0.007 | 8.92e-08 | 1.36e-05 | 13 / 24 | 0.002 |
| Phospholipid metabolism | 36 / 315 | 0.021 | 1.27e-07 | 1.63e-05 | 76 / 218 | 0.016 |
| Glycosaminoglycan metabolism | 26 / 183 | 0.012 | 1.38e-07 | 1.63e-05 | 29 / 88 | 0.006 |
| Gluconeogenesis | 15 / 66 | 0.004 | 2.36e-07 | 2.50e-05 | 10 / 26 | 0.002 |
| The citric acid (TCA) cycle and  respiratory electron transport | 28 / 238 | 0.016 | 1.84e-06 | 1.77e-04 | 34 / 67 | 0.005 |
| Metabolism of nucleotides | 28 / 256 | 0.017 | 7.05e-06 | 6.20e-04 | 45 / 141 | 0.01 |
| Glutathione conjugation | 13 / 68 | 0.005 | 9.31e-06 | 7.54e-04 | 6 / 14 | 0.001 |
| Cardiac conduction | 19 / 138 | 0.009 | 1.01e-05 | 7.67e-04 | 12 / 27 | 0.002 |
| Ion channel transport | 24 / 206 | 0.014 | 1.16e-05 | 8.22e-04 | 15 / 45 | 0.003 |
| Phase II - Conjugation of compounds | 28 / 265 | 0.018 | 1.31e-05 | 8.63e-04 | 32 / 72 | 0.005 |
| Activation of gene expression by  SREBF (SREBP) | 13 / 71 | 0.005 | 1.46e-05 | 9.04e-04 | 13 / 42 | 0.003 |
| Chondroitin sulfate/dermatan  sulfate metabolism | 13 / 73 | 0.005 | 1.94e-05 | 0.001 | 14 / 28 | 0.002 |
| Diseases associated with  glycosylation precursor biosynthesis | 9 / 37 | 0.002 | 3.58e-05 | 0.002 | 12 / 15 | 0.001 |
| Glycerophospholipid biosynthesis | 24 / 222 | 0.015 | 3.75e-05 | 0.002 | 45 / 133 | 0.01 |
| Respiratory electron transport, ATP  synthesis by chemiosmotic coupling,  and heat production by uncoupling  proteins | 19 / 153 | 0.01 | 4.03e-05 | 0.002 | 24 / 31 | 0.002 |
| Synthesis of IP3 and IP4 in the  cytosol | 9 / 39 | 0.003 | 5.34e-05 | 0.003 | 10 / 12 | 8.79e-04 |
| Biosynthesis of the N-glycan precursor (dolichol lipid-linked  oligosaccharide, LLO) and transfer to a nascent protein | 19 / 159 | 0.011 | 6.64e-05 | 0.003 | 17 / 63 | 0.005 |
| PKMTs methylate histone lysines | 10 / 51 | 0.003 | 7.99e-05 | 0.004 | 8 / 22 | 0.002 |
| Diseases of metabolism | 35 / 410 | 0.027 | 9.08e-05 | 0.004 | 48 / 190 | 0.014 |
