# Supplementary material for: Transcriptional, chromatin, and metabolic landscapes of LDHA inhibitor–resistant pancreatic ductal adenocarcinoma
Source: Front Oncol. 2022 Aug 2;12:926437. doi: 10.3389/fonc.2022.926437 (PMC9378957; doi:10.3389/fonc.2022.926437)
Supplement: Supplementary file 1 [file DataSheet_1.zip › Ziped tables/Table S17_Group 3 vs Group 4_Reactome analysis.docx]

**Table S17.** List of the top 25 most significantly altered metabolic pathways from the RNA-sequencing analysis performed in oxamate-resistant MIAPaCa2 cells treated with or without oxamate using the Reactome Pathway Analysis tool

| **Pathway Name** | **Entities** | | | | **Reactions** | |
| --- | --- | --- | --- | --- | --- | --- |
|  | **Found** | **Ratio** | **p-value** | **False Discovery Rate** | **Found** | **Ratio** |
| Cellular hexose transport | 2 / 28 | 0.002 | 4.07e-04 | 0.012 | 2 / 17 | 0.001 |
| Response of EIF2AK1 (HRI) to heme deficiency | 2 / 29 | 0.002 | 4.36e-04 | 0.012 | 2 / 20 | 0.001 |
| MECP2 regulates neuronal receptors  and channels | 2 / 32 | 0.002 | 5.30e-04 | 0.012 | 2 / 26 | 0.002 |
| ATF4 activates genes in response to  endoplasmic reticulum stress | 2 / 34 | 0.002 | 5.98e-04 | 0.012 | 1 / 7 | 5.13e-04 |
| SLC-mediated transmembrane transport | 4 / 421 | 0.028 | 8.46e-04 | 0.012 | 4 / 191 | 0.014 |
| PERK regulates gene expression | 2 / 42 | 0.003 | 9.08e-04 | 0.012 | 1 / 11 | 8.06e-04 |
| Transport of small molecules | 5 / 966 | 0.064 | 0.003 | 0.029 | 10 / 443 | 0.032 |
| Transcriptional Regulation by  MECP2 | 2 / 100 | 0.007 | 0.005 | 0.05 | 2 / 77 | 0.006 |
| Response of EIF2AK4 (GCN2) to  amino acid deficiency | 2 / 115 | 0.008 | 0.007 | 0.059 | 2 / 16 | 0.001 |
| The fatty acid cycling model | 1 / 8 | 5.31e-04 | 0.008 | 0.065 | 3 / 6 | 4.40e-04 |
| Unfolded Protein Response (UPR) | 2 / 155 | 0.01 | 0.012 | 0.065 | 1 / 94 | 0.007 |
| The proton buffering model | 1 / 11 | 7.30e-04 | 0.012 | 0.065 | 1 / 1 | 7.33e-05 |
| Transport of inorganic  cations/anions and amino  acids/oligopeptides | 2 / 167 | 0.011 | 0.013 | 0.065 | 2 / 75 | 0.005 |
| Cellular response to starvation | 2 / 176 | 0.012 | 0.015 | 0.065 | 2 / 28 | 0.002 |
| Erythrocytes take up oxygen and  release carbon dioxide | 1 / 16 | 0.001 | 0.017 | 0.065 | 1 / 6 | 4.40e-04 |
| Mitochondrial Uncoupling | 1 / 18 | 0.001 | 0.019 | 0.065 | 4 / 9 | 6.60e-04 |
| Glycerophospholipid catabolism | 1 / 18 | 0.001 | 0.019 | 0.065 | 1 / 7 | 5.13e-04 |
| Multifunctional anion exchangers | 1 / 19 | 0.001 | 0.02 | 0.065 | 1 / 6 | 4.40e-04 |
| Passive transport by Aquaporins | 1 / 21 | 0.001 | 0.022 | 0.065 | 2 / 8 | 5.86e-04 |
| Synaptic adhesion-like molecules | 1 / 23 | 0.002 | 0.024 | 0.065 | 3 / 8 | 5.86e-04 |
| O2/CO2 exchange in erythrocytes | 1 / 23 | 0.002 | 0.024 | 0.065 | 2 / 13 | 9.53e-04 |
| Erythrocytes take up carbon dioxide  and release oxygen | 1 / 23 | 0.002 | 0.024 | 0.065 | 1 / 7 | 5.13e-04 |
| Vitamin C (ascorbate) metabolism | 1 / 26 | 0.002 | 0.027 | 0.065 | 1 / 9 | 6.60e-04 |
| Basigin interactions | 1 / 26 | 0.002 | 0.027 | 0.065 | 1 / 10 | 7.33e-04 |
| Unblocking of NMDA receptors,  glutamate binding and activation | 1 / 27 | 0.002 | 0.028 | 0.065 | 4 / 5 | 3.66e-04 |
