# Supplementary material for: Transcriptional, chromatin, and metabolic landscapes of LDHA inhibitor–resistant pancreatic ductal adenocarcinoma
Source: Front Oncol. 2022 Aug 2;12:926437. doi: 10.3389/fonc.2022.926437 (PMC9378957; doi:10.3389/fonc.2022.926437)
Supplement: Supplementary file 1 [file DataSheet_1.zip › Ziped tables/Table S11_Group 3 vs Group 1_Reactome analysis.docx]

**Table S11.** List of the top 25 most significantly altered metabolic pathways from the RNA-sequencing analysis performed in parental (oxamate-sensitive) and oxamate-resistant MIAPaCa2 cells using the Reactome Pathway Analysis tool

| **Pathway Name** | **Entities** | | | | **Reactions** | |
| --- | --- | --- | --- | --- | --- | --- |
|  | **Found** | **Ratio** | **p-value** | **False Discovery Rate** | **Found** | **Ratio** |
| Metabolism | 210 / 3,643 | 0.242 | 1.11e-16 | 9.13e-14 | 351 / 2,251 | 0.165 |
| Transport of small molecules | 71 / 966 | 0.064 | 2.35e-14 | 9.67e-12 | 88 / 443 | 0.032 |
| SLC-mediated transmembrane  transport | 36 / 421 | 0.028 | 2.25e-09 | 6.17e-07 | 42 / 191 | 0.014 |
| Metabolism of lipids | 74 / 1,446 | 0.096 | 8.24e-08 | 1.69e-05 | 130 / 955 | 0.07 |
| Phospholipid metabolism | 27 / 315 | 0.021 | 2.32e-07 | 3.80e-05 | 57 / 218 | 0.016 |
| Metabolism of nucleotides | 23 / 256 | 0.017 | 8.40e-07 | 1.15e-04 | 36 / 141 | 0.01 |
| Transport of inorganic  cations/anions and amino  acids/oligopeptides | 18 / 167 | 0.011 | 1.11e-06 | 1.30e-04 | 18 / 75 | 0.005 |
| Ion channel transport | 20 / 206 | 0.014 | 1.39e-06 | 1.30e-04 | 17 / 45 | 0.003 |
| Metabolism of carbohydrates | 32 / 457 | 0.03 | 1.43e-06 | 1.30e-04 | 39 / 243 | 0.018 |
| Cardiac conduction | 16 / 138 | 0.009 | 1.76e-06 | 1.44e-04 | 10 / 27 | 0.002 |
| Glycosaminoglycan metabolism | 18 / 183 | 0.012 | 3.91e-06 | 2.90e-04 | 20 / 88 | 0.006 |
| TRP channels | 7 / 30 | 0.002 | 2.19e-05 | 0.001 | 2 / 4 | 2.93e-04 |
| Activation of gene expression by  SREBF (SREBP) | 10 / 71 | 0.005 | 3.12e-05 | 0.002 | 10 / 42 | 0.003 |
| Phase 0 - rapid depolarisation | 7 / 34 | 0.002 | 4.80e-05 | 0.003 | 2 / 2 | 1.47e-04 |
| Glycerophospholipid biosynthesis | 18 / 222 | 0.015 | 4.89e-05 | 0.003 | 41 / 133 | 0.01 |
| Synthesis of IP3 and IP4 in the  cytosol | 7 / 39 | 0.003 | 1.12e-04 | 0.006 | 9 / 12 | 8.79e-04 |
| Stimuli-sensing channels | 12 / 119 | 0.008 | 1.26e-04 | 0.006 | 8 / 28 | 0.002 |
| Amino acid transport across the  plasma membrane | 9 / 69 | 0.005 | 1.36e-04 | 0.006 | 9 / 36 | 0.003 |
| Hydrolysis of LPC | 5 / 18 | 0.001 | 1.50e-04 | 0.006 | 6 / 6 | 4.40e-04 |
| Regulation of cholesterol  biosynthesis by SREBP (SREBF) | 10 / 87 | 0.006 | 1.63e-04 | 0.007 | 10 / 52 | 0.004 |
| Muscle contraction | 16 / 213 | 0.014 | 2.95e-04 | 0.011 | 10 / 42 | 0.003 |
| Cholesterol biosynthesis | 9 / 77 | 0.005 | 3.04e-04 | 0.011 | 15 / 35 | 0.003 |
| Metabolism of steroids | 21 / 330 | 0.022 | 3.35e-04 | 0.012 | 36 / 245 | 0.018 |
| Interconversion of nucleotide di- and  triphosphates | 9 / 80 | 0.005 | 3.99e-04 | 0.014 | 13 / 33 | 0.002 |
| Synthesis of very long-chain fatty  acyl-CoAs | 7 / 51 | 0.003 | 5.55e-04 | 0.018 | 5 / 12 | 8.79e-04 |
