# Supplementary material for: Transcriptional, chromatin, and metabolic landscapes of LDHA inhibitor–resistant pancreatic ductal adenocarcinoma
Source: Front Oncol. 2022 Aug 2;12:926437. doi: 10.3389/fonc.2022.926437 (PMC9378957; doi:10.3389/fonc.2022.926437)
Supplement: Supplementary file 1 [file DataSheet_1.zip › Ziped tables/Table S13_Group 2 vs Group 1_Reactome analysis.docx]

**Table S13.** List of the top 25 most significantly altered metabolic pathways from the RNA-sequencing analysis performed in parental (oxamate-sensitive) MIAPaCa2 cells treated with or without oxamate using the Reactome Pathway Analysis tool

| **Pathway Name** | **Entities** | | | | **Reactions** | |
| --- | --- | --- | --- | --- | --- | --- |
|  | **Found** | **Ratio** | **p-value** | **False Discovery Rate** | **Found** | **Ratio** |
| Metabolism | 70 / 3,643 | 0.242 | 2.35e-11 | 1.06e-08 | 112 / 2,251 | 0.165 |
| Metabolism of carbohydrates | 22 / 457 | 0.03 | 2.77e-10 | 6.23e-08 | 36 / 243 | 0.018 |
| Transport of small molecules | 28 / 966 | 0.064 | 6.03e-08 | 8.74e-06 | 37 / 443 | 0.032 |
| Glucose metabolism | 11 / 140 | 0.009 | 9.33e-08 | 8.74e-06 | 13 / 50 | 0.004 |
| Glycolysis | 10 / 110 | 0.007 | 9.71e-08 | 8.74e-06 | 8 / 24 | 0.002 |
| Gluconeogenesis | 8 / 66 | 0.004 | 2.29e-07 | 1.72e-05 | 5 / 26 | 0.002 |
| SLC-mediated transmembrane  transport | 13 / 421 | 0.028 | 1.55e-04 | 0.01 | 14 / 191 | 0.014 |
| Manipulation of host energy  metabolism | 2 / 3 | 1.99e-04 | 3.73e-04 | 0.021 | 2 / 2 | 1.47e-04 |
| Cytosolic sulfonation of small  molecules | 5 / 74 | 0.005 | 6.65e-04 | 0.031 | 12 / 24 | 0.002 |
| Metabolism of water-soluble  vitamins and cofactors | 9 / 257 | 0.017 | 6.84e-04 | 0.031 | 13 / 144 | 0.011 |
| Classical Kir channels | 2 / 5 | 3.32e-04 | 0.001 | 0.041 | 1 / 1 | 7.33e-05 |
| Glycosaminoglycan metabolism | 7 / 183 | 0.012 | 0.002 | 0.061 | 10 / 88 | 0.006 |
| Basigin interactions | 3 / 26 | 0.002 | 0.002 | 0.064 | 3 / 10 | 7.33e-04 |
| Insulin receptor recycling | 3 / 28 | 0.002 | 0.002 | 0.07 | 2 / 6 | 4.40e-04 |
| Cellular hexose transport | 3 / 28 | 0.002 | 0.002 | 0.07 | 5 / 17 | 0.001 |
| Metabolism of vitamins and  cofactors | 10 / 382 | 0.025 | 0.003 | 0.077 | 14 / 206 | 0.015 |
| Ion channel transport | 7 / 206 | 0.014 | 0.003 | 0.077 | 7 / 45 | 0.003 |
| Potassium Channels | 5 / 107 | 0.007 | 0.003 | 0.077 | 6 / 19 | 0.001 |
| Phase II - Conjugation of compounds | 8 / 265 | 0.018 | 0.003 | 0.077 | 15 / 72 | 0.005 |
| Mitochondrial ABC transporters | 2 / 10 | 6.64e-04 | 0.004 | 0.087 | 2 / 2 | 1.47e-04 |
| Keratan sulfate biosynthesis | 3 / 37 | 0.002 | 0.005 | 0.105 | 5 / 9 | 6.60e-04 |
| Inwardly rectifying K+ channels | 3 / 38 | 0.003 | 0.005 | 0.105 | 3 / 7 | 5.13e-04 |
| ABC-family proteins mediated  transport | 5 / 122 | 0.008 | 0.006 | 0.105 | 6 / 27 | 0.002 |
| Transferrin endocytosis and  recycling | 3 / 39 | 0.003 | 0.006 | 0.105 | 2 / 11 | 8.06e-04 |
| Neuronal System | 11 / 489 | 0.032 | 0.006 | 0.105 | 25 / 216 | 0.016 |
