# Supplementary material for: Transcriptional, chromatin, and metabolic landscapes of LDHA inhibitor–resistant pancreatic ductal adenocarcinoma
Source: Front Oncol. 2022 Aug 2;12:926437. doi: 10.3389/fonc.2022.926437 (PMC9378957; doi:10.3389/fonc.2022.926437)
Supplement: Supplementary file 1 [file DataSheet_1.zip › Ziped tables/Supplementary_Material.FINAL.docx]

Supplementary Material

# Supplementary Tables Legend

**Table S1.** Differentially regulated genes identified from RNA-sequencing analysis between parental (oxamate-sensitive) MIAPaCa2 cells treated with and without oxamate.

**Table S2.** Differentially regulated genes identified from RNA-sequencing analysis between oxamate-resistant MIAPaCa2 cells treated with and without oxamate.

**Table S3.** Differentially regulated genes identified from RNA-sequencing analysis between parental (oxamate-sensitive) and oxamate-resistant MIAPaCa2 cells.

**Table S4.** Differentially regulated genes identified from RNA-sequencing analysis between oxamate-treated parental (oxamate-sensitive) and oxamate-treated oxamate-resistant MIAPaCa2 cells.

**Table S5.** Transcription factors identified by enrichment analysis performed by Enrichr (using ENCODE and ChEA consensus TF libraries) between parental (oxamate-sensitive) MIAPaCa2 cells treated with and without oxamate.

**Table S6.** Transcription factors identified by enrichment analysis performed by Enrichr (using ENCODE and ChEA consensus TF libraries) between parental (oxamate-sensitive) and oxamate-resistant MIAPaCa2 cells.

**Table S7.** Transcription factors identified by enrichment analysis performed by Enrichr (using ENCODE and ChEA consensus TF libraries) between oxamate-treated parental (oxamate-sensitive) and oxamate-treated oxamate-resistant MIAPaCa2 cells.

**Table S8.** List of metabolites altered between parental (oxamate-sensitive) and oxamate-resistant MIAPaCa2 cells treated with and without oxamate identified by a global untargeted metabolomics analysis using capillary electrophoresis time-of-flight mass spectrometry (CE-TOFMS).

**Table S9.** List of metabolic genes identified from RNA-seq data analyzed in parental (oxamate-sensitive) and oxamate-resistant MIAPaCa2 cells treated with or without oxamate.

**Table S10.** List of differentially regulated metabolic genes identified from RNA-seq data analyzed in parental (oxamate-sensitive) and oxamate-resistant MIAPaCa2 cells.

**Table S11.** List of the top 25 most significantly altered metabolic pathways from the RNA-sequencing analysis performed in parental (oxamate-sensitive) and oxamate-resistant MIAPaCa2 cells using the Reactome Pathway Analysis tool.

**Table S12.** List of differentially regulated metabolic genes identified from RNA-seq data analyzed in parental (oxamate-sensitive) MIAPaCa2 cells treated with or without oxamate.

**Table S13.** List of the top 25 most significantly altered metabolic pathways from the RNA-sequencing analysis performed in parental (oxamate-sensitive) MIAPaCa2 cells treated with or without oxamate using the Reactome Pathway Analysis tool.

**Table S14.** Transcription factors regulating the differentially expressed metabolic genes identified by enrichment analysis performed by Enrichr (using ENCODE and ChEA consensus TF libraries) between parental (oxamate-sensitive) and oxamate-resistant MIAPaCa2 cells.

**Table S15.** Transcription factors regulating the differentially expressed metabolic genes identified by enrichment analysis performed by Enrichr (using ENCODE and ChEA consensus TF libraries) between vehicle- and oxamate-treated parental (oxamate-sensitive) MIAPaCa2 cells.

**Table S16.** List of differentially regulated metabolic genes identified from RNA-seq data analyzed in oxamate-resistant MIAPaCa2 cells treated with or without oxamate.

**Table S17.** List of the top 25 most significantly altered metabolic pathways from the RNA-sequencing analysis performed in oxamate-resistant MIAPaCa2 cells treated with or without oxamate using the Reactome Pathway Analysis tool.

**Table S18.** List of differentially regulated metabolic genes identified from RNA-seq data analyzed in oxamate-treated parental (oxamate-sensitive) and oxamate-resistant MIAPaCa2 cells.

**Table S19.** List of the top 25 most significantly altered metabolic pathways from the RNA-sequencing analysis performed in oxamate-treated parental (oxamate-sensitive) and oxamate-resistant MIAPaCa2 cells using the Reactome Pathway Analysis tool.

**Table S20.** Transcription factors regulating the differentially expressed metabolic genes identified by enrichment analysis performed by Enrichr (using ENCODE and ChEA consensus TF libraries) between oxamate-resistant MIAPaCa2 cells treated with and without oxamate.

**Table S21.** Transcription factors regulating the differentially expressed metabolic genes identified by enrichment analysis performed by Enrichr (using ENCODE and ChEA consensus TF libraries) between oxamate-treated parental (oxamate-sensitive) and oxamate-treated oxamate-resistant MIAPaCa2 cells.

**Table S22.** List of significantly altered metabolic pathways identified from the integration of metabolomics and RNA-seq data analyses between parental (oxamate-sensitive) and oxamate-resistant MIAPaCa2 cells using the MetaboAnalyst 5.0 pathway analysis tool.

**Table S23.** List of significantly altered metabolic pathways identified from the integration of metabolomics and RNA-seq data analyses between vehicle- and oxamate-treated parental (oxamate-sensitive) MIAPaCa2 cells using the MetaboAnalyst 5.0 pathway analysis tool.

**Table S24.** List of significantly altered metabolic pathways identified from the integration of metabolomics and RNA-seq data analyses between vehicle- and oxamate-treated oxamate-resistant MIAPaCa2 cells using the MetaboAnalyst 5.0 pathway analysis tool.

**Table S25.** List of significantly altered metabolic pathways identified from the integration of metabolomics and RNA-seq data analyses between oxamate-treated parental (oxamate-sensitive) and oxamate-resistant MIAPaCa2 cells using the MetaboAnalyst 5.0 pathway analysis tool.
